# Determination of the essential number of motoneurons required to produce functionally useful hind limb locomotion

**DOI:** 10.4103/NRR.NRR-D-24-01350

**Published:** 2025-09-29

**Authors:** Zoltán Fekécs, Dénes G. Török, Gábor Márton, László Gál, Krisztián Pajer, Antal Nógrádi, Sándor Pintér

**Affiliations:** 1 Department of Anatomy, Histology and Embryology, Faculty of Medicine, University of Szeged, Szeged, Hungary; 2 Department of Traumatology, Semmelweis Hospital, Kiskunhalas, Hungary

**Keywords:** avulsion, gait analysis, hind limb denervation, kinematic analysis, minimally required motoneuron number, motoneuron, reinnervation, retrograde tracing, riluzole, ventral root reimplantation

## Abstract

Avulsion injury of one or more spinal ventral roots induces a critical loss of motoneurons, followed by irreversible locomotor function impairment ranging from inadequate limb movement to complete paralysis of the limb. Recent surgical techniques facilitate improvement of limb function, but it remains to be determined exactly how many motoneurons are needed to survive and grow new axons to achieve sufficient muscle reinnervation. The aim of this study was to determine the minimum motoneuron quantity required to reinnervate the denervated skeletal muscles of the limb and produce a functionally satisfactory locomotor pattern. Since none of the commercially available methods and equipment were able to provide a quantifiable and in-depth analysis of the motor pattern of the entire hind limb, we have developed and applied a sensitive movement recording and analyzing system in order to determine the threshold of satisfactory functional reinnervation; we combined video-based footprint analysis and hind limb motion analysis to achieve a new and reliable assessment. Sprague–Dawley rats underwent a lumbar 4–5 ventral root avulsion, and their L4 ventral roots were subsequently reimplanted. The animals received different doses of riluzole treatment in order to rescue incremental numbers of the damaged motoneuron pool. We were able to assess one rear-view and six lateral parameters of the hind limb movement pattern by measuring specific joint angles, footprint, and gait parameters in single video frames. Four months after the operation, we performed Fast Blue retrograde tracing to label and count the reinnervating motoneurons. We then compared the numbers of reinnervating motoneurons and the functional improvement. Our results confirmed a strong relationship between functional restoration of the original movement pattern and morphological reinnervation; approximately 30% of the original motor pool was able to produce a useful locomotor pattern. We believe that our knowledge of the minimal motoneuron numbers required to reinnervate target muscles may help plan the segmental redistribution of the motoneuron pools for reinnervation surgeries.

## Graphical abstract

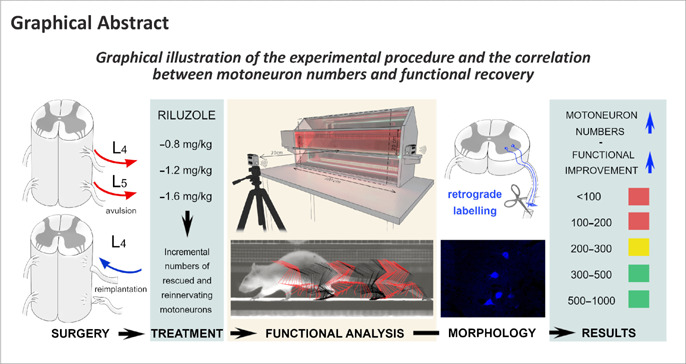

## Introduction

Motoneuron loss often results in irreversible locomotor function impairment after injuries such as avulsion of one or more ventral roots (Nemeth et al., 2024). The motor system appears to be over-insured: studies have proved that 60%–70% of the original motor pool can provide satisfactory and coordinated limb movement in a rat model (Doherty, 2003; Pintér et al., 2010). Recent studies investigating neurodegenerative diseases such as amyotrophic lateral sclerosis (ALS) and spinal muscular atrophy (SMA) have described that weight-bearing muscles can be preserved even in advanced states of the disorders (Bowerman et al., 2018; Hepple, 2018; Fogarty et al., 2021; Castro et al., 2023). It has also been reported that following peripheral nerve injuries, partially denervated muscles receive innervation by the expansion of motor units, which is another proof of the system’s plasticity (Sławińska et al., 1996). These data suggest that the correlation between morphological innervation and functional locomotor capacity of the limb is not fully linear along the entire scale of available motoneurons. The Medical Research Council (MRC) scale for muscle strength and the Modified MRC scales are used for the clinical grading of limb musculature. Generally, an M3 grade movement strength is able to act against gravity, an M4 grade movement strength is capable of acting against some resistance, while an M5 grade indicates the normal strength of the muscles (Barrie et al., 2004; Yang et al., 2015; Mathews et al., 2017). In cases of peripheral neurosurgical reconstruction of damaged extremities in humans, the availability of donor motor nerves is very limited (Dibble et al., 2019; Wilson, 2019). A good alternative source is the sharing of a motor nerve between the donor muscles and new targets requiring fresh motor innervation (Wei et al., 2014; Čebron et al., 2021; Eli et al., 2021; Laohaprasitiporn et al., 2024). Therefore, it is critical to achieve a distribution of motor axons between donor and new target muscles in a way that does not jeopardize the motor function of either muscle group (Dewolf et al., 2019; Avaltroni et al., 2024). Thus, the minimally required motoneuron quantity for efficient (M4–5) muscle strength would be essential information for morphological reinnervation after motor function loss.

An intact L4 motor pool in humans aged 13 to 40 consists of an average of 12,000 motoneurons (Tomlinson and Irving, 1977). In contrast, the intact rat L4 spinal segment contains approximately 1200 motoneurons (Nógrádi and Vrbová, 2001), thus only a very careful proportional comparison may be possible between the rat and human motoneuron pools with all the anatomical differences taken into consideration. It should also be considered that rodents are quadrupedal animals, while humans display a bipedal locomotion pattern with a completely different muscle tone and force distribution pattern. Compared with quadrupedal animals, it should also be noted that humans possess a number of muscles that perform delicate and very well-planned movements, which require more motoneurons with small motor units. This is in sharp contrast with muscles performing rough and bulk movements. To match motoneuron numbers with functional abilities in rodents, a sensitive locomotor pattern analysis system is needed, since the detection of minor changes induced by a limited number of motoneurons is an essential requirement. In our earlier studies, we successfully applied our custom-made multi-parametric video-based locomotor system (Török et al., 2022), which also proved to be sensitive enough to observe minor changes in the locomotor pattern.

Avulsion injury occurs at the boundary of the central and the peripheral nervous systems. Without therapy, this type of injury leads to the death of the vast majority of motoneurons (Nógrádi et al., 2007; Ter Wengel et al., 2018; Zhang et al., 2019; Adegeest et al., 2024). Earlier studies have provided evidence that various doses of riluzole successfully rescue motoneurons with avulsed axons (Gloviczki et al., 2017; Bolandghamat and Behnam-Rassouli, 2020; Fang et al., 2020; Pajer et al., 2023). Our earlier papers showed the efficacy of riluzole even in special cases, such as delayed administration starting on Day 10 after avulsion injury (Nógrádi et al., 2007) or in delayed reconstruction models (Gloviczki et al., 2017). Riluzole (2-amino-6-trifluoromethoxybenzothiazole) is a neuroprotective compound that blocks voltage gated Na^+^ and Ca^2+^ channels and activates K^+^ channels. Moreover, it is able to inhibit presynaptic glutamate release (Doble, 1996). Riluzole is the only drug proven to have some efficacy in a subset of ALS patients, and is still the only FDA-approved drug used for the systemic treatment of ALS (Wijesekera and Leigh, 2009). The side effects of riluzole are usually tolerable, thus it can be administered for relatively long periods. Previous studies have also proven that the rescued motoneurons were able to grow their axons towards their target muscles, resulting in nearly complete reinnervation (Chai et al., 2000; Pajer et al., 2014, 2015; Huang et al., 2022a, b). Therefore, it was thought that ventral root avulsion combined with a progressively increased dose of riluzole could establish a good model for studying this clinically relevant question.

The aim of the present study was to investigate the minimally required number of motoneurons in a rat model which is able to produce weight-bearing locomotion after a clinically relevant ventral root avulsion injury followed by ventral root reimplantation. The evaluation of the locomotor pattern coupled with the morphological reinnervation may provide information on the regenerative process itself.

## Methods

### Animals

**Figure 1:**
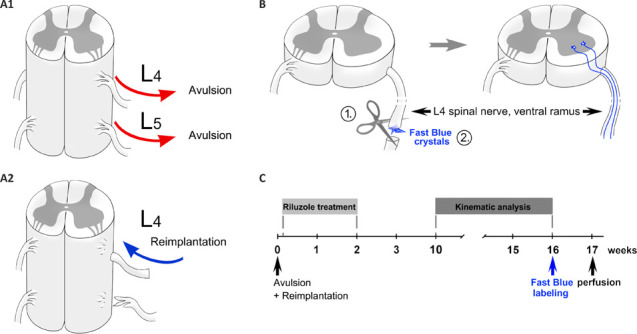
Schematic diagrams show the surgical setup and a time-scaled overview of the experiment. (A) The lumbar 4–5 (L4–L5) ventral roots were pulled out of the spinal cord (A1) and the L4 root was immediately reimplanted (A2) in a lateral position of the caudal L4 segment. (B) Sixteen weeks after the avulsion and reimplantation, the L4 spinal nerve was cut and Fast Blue fluorescent retrograde tracer was applied to the proximal stump. (C) The timeline of the riluzole treatment, the video recording and the retrograde tracing is shown (*n* = 30).

A total of 39 female Sprague–Dawley rats (Biological Services, University of Szeged, 170–220 g body weight, 1.5–2 months old) were used in this study. We used female rats only according to our previous studies (Török et al., 2022). The animals were kept at 24°C, 50% humidity with a 12-hour light-dark cycle set in a Conventional Housing Facility (CON). The cages had a base surface of 2000 cm^2^ and 2 or 3 rats shared one cage. The animals received water and food *ad libitum*. The experiments were carried out with the approval of the Committee for Animal Experiments, University of Szeged regarding the care and use of animals for experimental procedures and under the auspices of the National Food Chain Safety Office, Hungary (NFCSO, animal license No.: I./1569/2019). All the procedures were performed according to the corresponding animal legislation of the EU (European Convention for the Protection of Vertebrate Animals used for Experimental and other Scientific Purposes 01/01/1991). Nine animals were used in a preliminary study to map by retrograde labeling the location and distribution of the innervating motoneurons of three hind limb muscles important in moving the ankle joint. This was needed to prove whether the chosen spinal segments were appropriate for the avulsion injury to be performed. Accordingly, thirty animals were used for left lumbar 4 and 5 (L4–L5) ventral root avulsion and immediate reimplantation of the L4 root. From this pool of operated animals, 24 received various doses of riluzole after the operation, while six animals remained untreated (control animals). The animals survived for 16 weeks, and biweekly kinematic analysis was carried out during the survival period (**Figure 1**).

### Preliminary retrograde tracer injection to map the pool of motoneurons innervating the extensor digitorum longus, tibialis anterior, and soleus muscles

To determine the exact contribution of the L3, L4 and L5 spinal segments to the innervation of the main hind limb muscles in Sprague–Dawley rats (*n* = 9), Fast Blue fluorescent tracer solution (2%, dispersed in distilled water, Illing Plastics GmbH, Gross-Umfeld, Germany) was pressure-injected into the tibialis anterior (TA) (2 × 3 μL), the extensor digitorum longus (EDL) (2 μL) and the soleus (SOL) (2 μL) muscles (*n* = 3 each muscle) on the left side of the hind limb. These muscles are essential for the dorsiflexion (TA and EDL) and plantarflexion (SOL) of the ankle joint in rats. The extent of the L4 spinal cord segment was determined through the use of Diamidino-Yellow tracer (Illing Plastics GmbH) applied to the cut proximal stump of the L4 spinal nerve 3 days after labeling with Fast Blue (**Figure 1**). The animals were perfused one week after Fast Blue administration.

### Surgical procedures

All the operations were carried out under deep ketamine-xylazine anesthesia [ketamine hydrochloride (Ketavet, Dr. E. Graeub AG, Bern, Switzerland, 110 mg/kg body weight, i.p.); xylazine (Rompun, Bayer HealthCare, Bayer Austria GmbH., 12 mg/kg body weight, i.p.)] taking sterile precautions. The animals were given saline (0.9%; 5 mL) to prevent dehydration, and also received meloxicam (Metacam; 0.5 mg/kg body weight, Boehringer Ingelheim Vetmedica, Duluth, GA, USA for 3 days) postoperatively. Efforts were made to minimize the number of animals used.

Laminectomy was performed at the level of T13–L1 vertebra, the dura was opened, the spinal cord and the ventral roots were exposed, and the left L4–L5 ventral roots were pulled out, leaving the dorsal roots intact. The avulsion of both (L4–L5) ventral roots was essential as a major part of the motor innervation of SOL appeared to derive from the L5 segment in rats. Then the free end of the avulsed L4 ventral root was gently inserted into the dorsolateral part of the caudal L4 spinal segment close to the L4 motor pool (Chai et al., 2000). The reimplantation of only the L4 root along with the L5 root avulsion made it possible to reinnervate both the ankle dorsiflexors (extensors) and plantarflexors. In this way, we were able to determine the proportion of the motoneuron pool required to produce a reasonable movement pattern. The spinal cord was covered with the remaining dura, the wound was closed in two layers, and the animals were allowed to recover.

### Riluzole treatment

The animals (*n* = 24) were treated with riluzole (2-amino-6-trifluoromethoxy-benzothiazole, a kind gift from Tocris Cookson Ltd., Langford, UK) for 2 weeks. The treatment started on the day after the surgery, and then the drug was administered intraperitoneally every other day for 2 weeks. To induce various surviving and reinnervating motoneuron numbers in the L4 pool, we used incremental doses of riluzole dissolved in sterile physiological saline: 0.8 mg/kg (*n* = 6), 1.2 mg/kg (*n* = 12; this figure is discussed in detail in the Results section), and 1.6 mg/kg (*n* = 6). The timeline protocol is based on the riluzole treatment strategy described in our earlier papers (Gloviczki et al., 2017).

### Retrograde labeling to map surviving neurons

Sixteen weeks after the avulsion injury, all the animals were deeply anesthetized with the combined ketamine-xylazine method. On the operated side, the ventral ramus of the L4 spinal nerve was sectioned and the proximal stump of the nerve was covered with few crystals of Fast Blue (**Figure 1B**). Five days after the application of the fluorescent tracer, the animals were re-anesthetized as described above and perfused transcardially with physiological saline followed by 4% paraformaldehyde in 0.1 M phosphate buffer (pH 7.4). The lumbar part of the spinal cord with the reimplanted ventral root was removed and kept in the same fixative overnight at 4°C. Then the tissues were cryoprotected in 30% sucrose in azide-PBS until further use.

### Cell counting

The quantity of retrogradely labeled motoneurons was determined in 25 μm-thick serial cryostat cross sections (Leica CM-1850 cryostat, Leica GmbH, Wetzlar, Germany) under an Olympus BX50 fluorescence microscope (Olympus Ltd., Tokyo, Japan). To avoid double counting of motoneurons present in consecutive sections, the retrogradely labeled neurons were mapped with the aid of an Olympus (Olympus, Tokyo, Japan) drawing tube, and their locations were compared with those of the labeled neurons in the previous section. All sections from the L4 motoneuron pool and few more from the upper part of L5 (until labeled motoneurons were found) were used.

### Kinematic analysis

#### Equipment and setup

**Figure 2:**
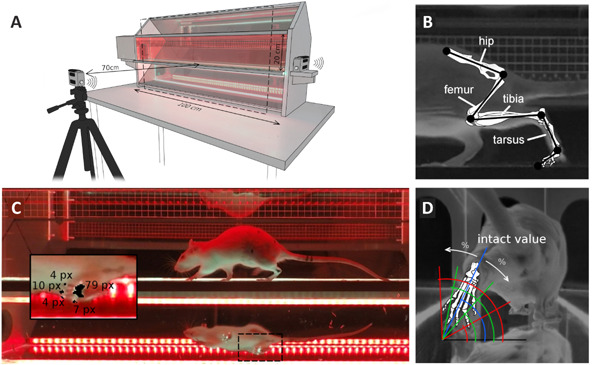
Different aspects of the kinematic analysis in detail. (A) The dimensions of the runway, along with the positioning of the cameras, were planned carefully to gain the most reliable data from the recorded videos. (B) Projection of the bones and the joints of the rat hind limb used in our measurements is shown. (C) The footprint area was measured at the moment when the contralateral limb was in mid-swing position. The pixels were selected automatically by color temperature (inset) and summed. (D) The measurement of the rear-view parameters is based on the angle enclosed by a selected bone and the floor plate (here the metatarsus is shown at the first moment of the swing phase). The intact value is displayed in blue, while green and red angles represent the deviations followed by moderate or serious impairments, respectively. It is important to note that deviations may occur in both directions (white arrows).

For kinematic analysis, we used our custom-made video-based kinematic analysis system as we described in detail earlier (Török et al., 2022). Briefly, it consists of a transparent plexiglass runway combined with a mirror system (**Figure 2A**) to observe and record the movements and positions of the contralateral limb. The runway was lit by red LEDs (650 nm) with their beams guided into the plexiglass side walls, and by green LEDs (530 nm) built into the floor plate (**Figure 2A** and **C**). The rats were trained preoperatively to walk from one end of the runway to a dark shelter at the other end. Small pieces of cereal were provided in the shelter to encourage the animals to cooperate during locomotor recordings. The locomotor pattern of the rats was recorded with high-resolution, high-speed digital cameras (GoPro Hero 5+ Black Edition, [1920 × 1080 pixels, 120 frames/s], GoPro Inc., San Mateo, CA, USA) from both lateral and posterior aspects simultaneously. The lateral camera was placed at a distance of 70 cm from the runway to record 3–4 step cycles within each run. Each animal had to produce a minimum of three runs suitable for locomotor analysis.

#### Locomotor test

After the avulsion and reimplantation surgery, a 10-week-long recovery period was allowed, after which video-recording sessions were performed biweekly. These time settings were chosen in accordance with our previous studies (Török et al., 2022). The shaved skin of the animals was marked with permanent black ink above the bony prominences of the major joints (**Figure 2B**). We recorded three runs of each animal in every session and examined five steps complying with two criteria: one similar step was required before and after the investigated step, and the head of the animal had to point in the direction of its travel. First, a digital fish-eye effect elimination was carried out (GoPro Studio software, GoPro Inc.) on the appropriate video sequences, and then the corrected sequences were split into single frames using VirtualDub (Avery Lee) software (https://www.virtualdub.org/). The selected frames where the animals were in a defined phase of motion were used for measurement using ImageJ 1.51j8 software (National Institutes of Health, Bethesda, MD, USA). The ImageJ software can provide the Cartesian coordinates of the plotted joint points. Conversion of the coordinates into vectors and subsequently into angles was performed using algorithms in the Microsoft Excel (Microsoft Corp., Redmond, WA, USA) environment and developed in our laboratory (Török et al., 2022). As growing intact rats displayed an ever-changing locomotor pattern, we had to correlate our recordings of injured animals with an earlier database involving intact animals (Török et al., 2022). This corrected comparison allowed us to carry out measurements in a wide range of step durations, step lengths and weight.

#### Gait parameters

Altogether, the evaluation of 7 gait parameters was carried out. All the parameters were described in detail in our earlier paper concerning locomotor analysis (Török et al., 2022). The parameters recorded from the lateral aspect were the following: toe-off angle (TOA), ankle flexion (AF), knee flexion (KF), lateral placing (LP), ankle lifting (AL), and knee lifting (KL). Metatarsus-surface angle (MSA) was recorded from the rear aspect. We also carried out footprint area analysis.

### Statistical analysis

No statistical methods were used to predetermine sample sizes; however, our sample sizes are similar to those reported in previous publications (Török et al., 2022). An arithmetic mean was used in the preliminary innervation pattern study. The repeated measures analysis of variance with the least significant difference *post hoc* test was applied for the statistical comparison of data from all groups (*P* ≤ 0.05). All the statistical analyses were carried out with RStudio 2022.12.0 (Posit Software, PBC, Boston, MA, USA).

## Results

### Segmental innervation of tibialis anterior, extensor digitorum longus, and soleus muscles in intact rats

**Figure 3:**
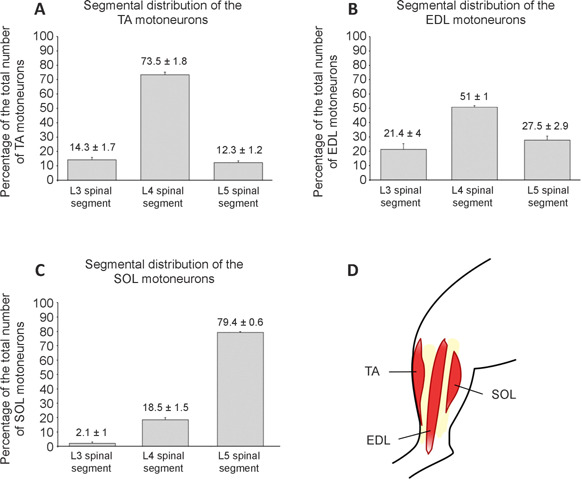
The segmental innervation pattern of the main muscles of the Sprague–Dawley rat hind limb is shown (*n* = 9). Main ankle dorsiflexors are tibialis anterior (TA; A) and extensor digitorum longus (EDL; B), and the main plantarflexor is soleus (SOL; C). Data are expressed as the mean ± SEM. (D) Schematic arrangement of the examined muscles. An arithmetic mean was used in the preliminary innervation pattern study.

To map the normal segmental innervation pattern of the most important ankle dorsi- and plantarflexors, we performed retrograde labeling from these muscles. The results are shown in **Figure 3**. In Sprague–Dawley rats, the TA is innervated by 101 motoneurons (±8.9 SEM); most of them are located from the L4 spinal segment (73.5% ± 1.8% SEM), and only a few motoneurons from the L3 and L5 segments contributed to its innervation (14.3% ± 1.7%, *vs*. 12.3% ± 1.2% SEM, respectively). This also proved to be partially true for the EDL (total: 76 motoneurons ±5.7 SEM; 51% ± 1% SEM in the L4 spinal segment), with the clear difference that the L3 and L5 spinal segments were responsible for a relatively larger innervation ratio than in the case of TA (21.4% ± 4%, *vs*. 27.5% ± 2.9% SEM, respectively). In contrast, the soleus muscle was found to receive its innervation mostly from the L5 spinal segment (total: 74 motoneurons ±3.9 SEM; 79.4% ± 0.6% SEM in L5) with a small number of motoneurons originating from the L4 segment (18.5% ± 1.5% SEM) and with very few innervating motoneurons from the L3 segment (2.1% ± 1% SEM). These data suggest that an L4–5 ventral root avulsion followed by the reimplantation of the L4 ventral root may result in a severe functional impairment of both the ankle dorsiflexors (receiving approximately 82% of their innervations from the L4–5 segments) and ankle plantarflexors (mainly innervated by L5 motoneurons).

### Correlation of reinnervating motoneuron numbers with functional outcome

**Figure 4:**
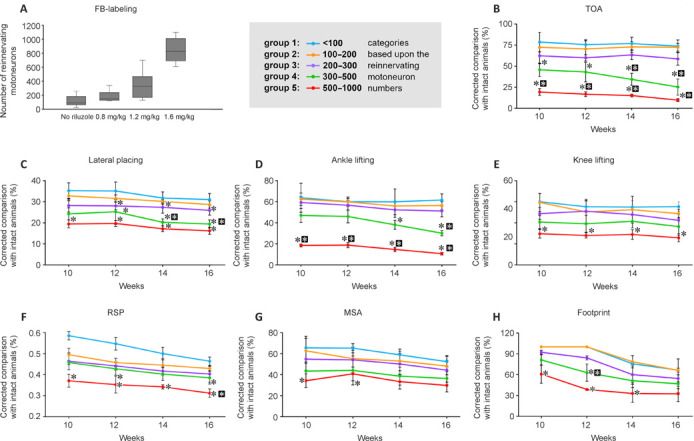
The diagrams show the results of the retrograde labeling (A) and the measured kinematic parameters in groups with different numbers of reinnervating motoneurons from the 10^th^ to the 16^th^ postoperative week (B–H, each color represents a secondary group as it is shown in A, right panel). (A) The secondary groups are based on the different ranges of motoneuron numbers and not on riluzole doses. Please note the standard deviation (SEM) in the group treated with 1.2 mg/kg riluzole (Group 1: *n* = 6, Group 2: *n* = 6, Group 3: *n* = 7, Group 4: *n* = 6 and Group 5: *n* = 5). (B–H) The deviation from the intact data (%) is shown in all the kinematic parameters. Significant differences between Group 1 (< 100 reinnervating) motoneurons) and any other group are indicated with simple asterisks (*P* < 0.05, mean ± SEM), while a white asterisk within a black frame indicates a significant difference between the adjacent groups (e.g., Group 4 to Group 3 or Group 5 to Group 4, *P* < 0.05, mean ± SEM). The repeated measures analysis of variance with the least significant difference post hoc test was applied for the statistical comparison of data from all groups.

To determine the critical number of motoneurons required to produce satisfactory function in weight-bearing muscles, we tried to create several groups of animals with various incremental numbers of reconnecting L4 motoneurons after L4–5 ventral root avulsion and L4 reimplantation. The first diagram of **Figure 4A** shows the number of reinnervating motoneurons in the L4 spinal segment obtained with different doses of riluzole treatment. This was intended to be achieved by administering increasing doses of riluzole to rescue hopefully incremental numbers of motoneurons in our model. In this present experimental setup, we observed that riluzole acts unpredictably up to and including a dosage of 1.2 mg/kg. Consequently, to observe this phenomenon in detail, we decided to expand the size of this group to *n* = 12. The motoneuron numbers of this expanded group still varied relatively widely (85 to 638 motoneurons); however, these numbers matched the functional performance of the individual animals. Since our goal was to find the correlation between the quantity of reinnervating motoneurons and functional improvement, we decided to ignore the applied doses of riluzole inducing different degrees of reinnervation and created new experimental groups after counting the FB^+^ motoneurons based on the number of reinnervating motoneurons (**Figure 4A**, right panel). Accordingly, 5 groups were created as follows: Group 1: less than 100 reinnervating motoneurons (*n* = 6, this group was identical with the primary control group) Group 2: 100–200 reinnervating motoneurons (*n* = 6), Group 3: 200–300 reinnervating motoneurons (*n* = 7), Group 4: 300–500 reinnervating motoneurons (*n* = 6), and Group 5: 500–1000 reinnervating motoneurons (*n* = 5; **Figure 4A**). We used these secondary groups for further statistical investigations as well.

### Kinematic analysis

**Figure 5:**
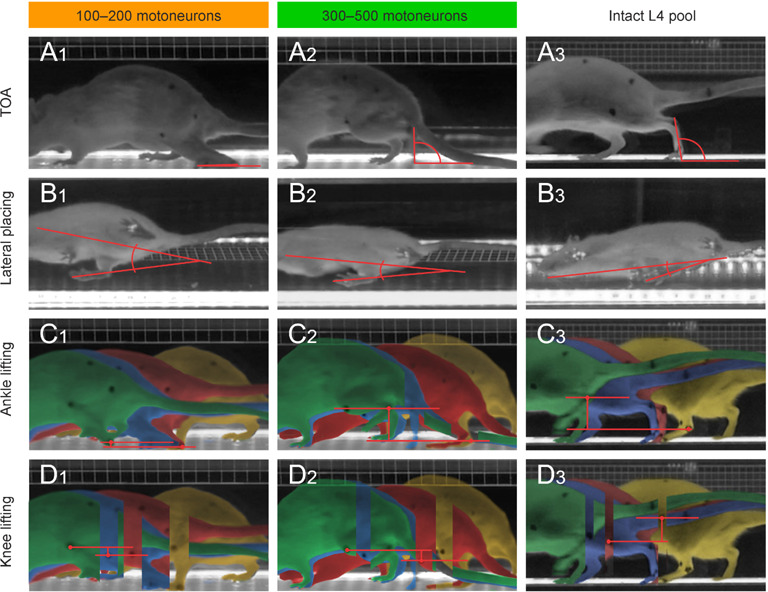
Representative images of the kinematic parameters are taken from the last video recording session (week 16). The columns represent the animals with different reinnervating motoneuron numbers (100–200 motoneurons = Group 2, *n* = 6; 300–500 motoneurons = Group 4, *n* = 6; intact L4 pool = Group 5, *n* = 5). (A1–A3) Toe-off angle (TOA) varied widely among the animals with different motoneuron numbers, thus it was believed to be a characteristic indicator of reinnervation. (B1–B3) Lateral placing typically appeared larger in animals with lower motoneuron numbers. (C1–C3) The general appearance of ankle lifting is shown. The four compared frames of the step cycle are shown in different colors (the first moment of the stance phase in yellow, the contralateral mid-swing phase in red, the last moment of the stance phase in blue and the ipsilateral mid-swing phase in green). (D1–D3) The knee lifting parameter is measured in the same way as ankle lifting. These parameters were typically smaller in animals with lower motoneuron numbers.

**Figure 6:**
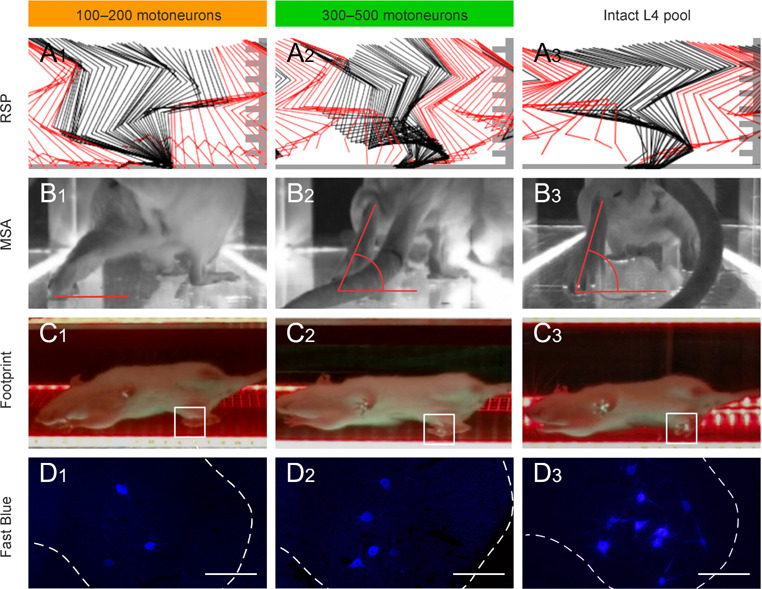
Representative images of the kinematic parameters are taken from the last video recording session (week 16). (A1–C3) and from the retrograde labeling. The columns represent the animals with different reinnervating motoneuron numbers (100–200 motoneurons = Group 2, *n* = 6; 300–500 motoneurons = Group 4, *n* = 6; intact L4 pool = Group 5, *n* = 5). (A1–A3) The black lines within the figures represent the number of frames belonging to the stance phase; the red ones belong to the swing phase. The ratio of the stance and swing phases (RSP) has been calculated and shown in Figure 4F. (B1–B3) The representative images show the metatarsus-surface angle (MSA) rear-view parameter in the secondary experimental groups. (C1–C3) The footprint is seen as a light green illuminated area. The degree of weight-bearing is reflected in the measured footprint area. (D1–D3) Representative images of the reinnervating motoneurons within the lumbar 4 spinal segment are shown.

We observed that from the 10th postoperative week onwards, even the animals with very limited range of movement (Group 2) presented detectable plantar stepping, while dorsal stepping was only visible in the control group (Group 1; **Figure 5A**) during the first weeks of regeneration. Lower motoneuron numbers resulted in visible difficulties in weight bearing, while the gait characteristics of the animals with the highest achieved reinnervating motoneuron numbers closely approached the gait features of the intact animals (**Figures 4B–H**, **5A–D**, and **6A–C**). The time course of the regeneration varied among the individual animals and also among different parameters, which can be considered as a benefit of the multi-parametric evaluation. Data from each secondary group was compared with the intact database by applying a correction method as previously described (Török et al., 2022). The result is shown in **Figure 4**. TOA, ankle flexion, and lateral placing parameters displayed significant improvement during the last part of the innervation process (from Week 12 to Week 16) in both groups 4 and 5 (300–500 *vs.* 500–1000 reinnervating motoneurons, respectively). Particular improvement was seen in Group 4 animals in all three parameters from Week 14 onwards. Group 5 showed significant changes in knee lifting, RSP, and footprint from Week 10, however, the footprint area parameter displayed by Group 4 animals showed significant improvement in the 12^th^ postoperative week. Only occasionally was a significant improvement of the MSA parameter (**Figure 6B**) detected as early as in Weeks 10 and 12 in Group 5.

## Discussion

In this study, we investigated whether it was possible to determine a minimum motoneuron quantity for the reinnervation of weight-bearing hind limb muscles that was able to produce functionally meaningful walking patterns in rats. We found that when only approximately 10% of the motoneurons in the L4 spinal segment were available to the reinnervation of both flexor and extensor hind limb muscles, an unstable locomotor pattern could be achieved, although 20% of the total motoneuron pool was already able to produce some meaningful general locomotor patterns. It should be noted, however, that this limited muscle innervation pattern yielded a measurable locomotor pattern only in the non-vital hind limb muscles in our experiments.

It should be considered that very low motoneuron numbers can result in the total deterioration of the pool, as very large motor units do not survive for long because they are both functionally and morphologically overstretched. This is seen in ALS cases, where the patients lose at least 20% of their original motoneuron pool in the presympotomatic phase (Mitsumoto et al., 2022), but the number of large-diameter axons is reduced by only 20% or less by the final demise of the disease (Hanyu et al., 1982). Similar observations have been reported as a late effect of poliomyelitis, referred to as post-polio syndrome (Sáinz et al., 2022). Paralytic poliomyelitis survivors may develop further muscle weakness and fatigue many years after the acute illness, most likely due to the deterioration of their already oversized motor units. This was precisely described by Wiechers and Hubell (1981) as a degeneration of the affected motor units. It has been clearly demonstrated that human motor axons can reinnervate up to 5 times their original territory by axonal sprouting within the skeletal muscle, but this figure also reflects the end of the reinnervating capacity of the motor units (Brown et al., 1981; Rimer et al., 2019; Blasco et al., 2020; Lee et al., 2023). Indeed, significant changes in the functional parameters could be observed in animals in which 30 to 40% of the motoneurons were able to reinnervate target muscles (i.e., 4 out of 7 parameters showed significant changes in Group 4 [300–500 motoneurons]). Therefore, it can be argued that the motor pool size is in concordance with the stability of reinnervation. With higher doses of riluzole, 75% of the motoneurons could be rescued in extreme cases, and this level of motoneuron survival was accompanied by a remarkable functional outcome, producing functional data nearly as good as those of intact animals.

Considering the determination of the minimum number of the available useful motoneurons, several questions could be raised, such as the functional types of muscles (slow muscles are less sensitive to denervation according to Iyer et al. (2021)), the size and number of motor units associated with a muscle, and the type of use of muscle. Smaller muscles performing delicate movements require more motoneurons with precise innervation, whereas bulk mass muscles likely tolerate motoneuron loss better (Sobue et al., 1981; Terzis and Barmpitsioti, 2012). It is also very likely that the distribution of reinnervating fibers changes among the denervated muscles. In our model, the soleus muscle loses 80% of its original innervating axons, but it is likely that more than 20% of motor end plates will be innervated by the reinnervating L4 motoneurons due to the availability of a large number of denervated end plates attracting freshly in-growing motor fibers. Moreover, the newly formed motor units will definitely be larger. Therefore, the functional improvement of the plantarflexion has to be partially supported by the gastrocnemius and peroneus muscles, which are mostly innervated by motoneurons in the L5–S1 segments (Weeks and English, 1985). This phenomenon does not weaken our model since it is similar to the clinical situation. Earlier, we found that a satisfactory weight-bearing gait in rodents can likely be matched to a human M4 movement (full range motion against gravity and resistance with decreased strength; Doherty, 2003; Pintér et al., 2010), which is a sufficient condition to be achieved. Our present data suggest that approximately 500 motoneurons of the L4 pool could produce M4 movement in rodents, which roughly corresponds to 42% of the intact pool. However, we need to emphasize that these numbers may be related to the type and extent of the injury and the duration of the denervated state of the muscles. Moreover, it is evident that the result of the reinnervation in the case of quadrupedal gait of rodents and other animals is not comparable to the bipedal movement of humans. In the latter case, the whole upper body weight is loaded onto the pelvis and lower limbs, while in rodents and other quadrupedal mammals, the weight of the body is supported by a different mechanism and four limbs.

Terzis and Barmpitsioti (2012) suggest the use of M2^+^ or M3^–^ categories for the type of motion which can act partially against gravity, which we believe best describes the performance of our Group 3 animals (200–300 reinnervating motoneurons). The gait analysis does not provide direct information about the strength of the movements, although details of certain parameters, such as footprint area and knee lifting, allowed us to evaluate the level of weight-bearing and strength. We believe that 300 to 500 reinnervating motoneurons (Group 4) can provide a reliable M3-like movement in this quadrupedal model, while 500–1000 motoneurons are enough to produce an M4 gait. Control animals with fewer than 100 reinnervating motoneurons (Group 1 = L4–5 avulsion and L4 reimplantation only) showed an M2 movement (they were able to execute limited-range movements with their hind limbs). Some of these animals showed slight permanent contracture in their hind limb joints at the end of week 16. In our view, the cases where an individual muscle or muscle groups are reinnervated by only 20% of their original set of motor axons carry a risk of late deterioration of the greatly enlarged motor units.

Our experience with the dosage of riluzole suggested that the kinetics of riluzole do not produce a linear correlation between the dose and the number of surviving motoneurons. However, several clinical spinal cord injury (SCI) studies suggest that below a certain dose, depending upon specific administration circumstances, riluzole remains ineffective (Grossman et al., 2014; Fehlings et al., 2016, 2023; Chow et al., 2023). It has also been reported that higher riluzole doses (e.g., 8 mg/kg) do not necessarily induce better outcomes (Zhou et al., 2019). Moreover, over a critical dosage, riluzole may also produce systemic side effects (Wu et al., 2010). It is also important to note that the effective dose-range of riluzole also depends on the motoneuron disorder; a higher dose is reportedly more efficient in ALS compared to SCI (Lacomblez et al., 1996; Saitoh and Takahashi, 2020). In our previous studies, we also used lower dose of riluzole to induce a limited innervation (0.8 mg/kg riluzole was used in an L4 avulsion and reimplantation model; Török et al., 2022); however, the surgical setup was different. In this study, we found that a dose limit to induce innervation was around 1.2 mg/mL, so we suggest that riluzole may have switch-like kinetics with a typical “turn on” mechanism. This efficiency baseline was also mentioned in human clinical trials, based on blood concentration measurements (Fehlings et al., 2016). In conclusion, it can be assumed that riluzole acts with near-linear kinetics only within a dose range, and this range of concentration likely depends on the type of SCI (Zhou et al., 2019).

We believe that our knowledge of the minimal motoneuron numbers required to reinnervate target muscles may help plan the segmental redistribution of the motor pools for reinnervation surgeries. This means that target muscles have to receive reliable, long-lasting innervation in order to maintain long-term function, and the number of motor axons transferred from the donor muscle to a new target muscle has to provide satisfactory innervation (at least 30% of the original motoneuron pool, or approximately 4000 motoneurons in the case of the L4 segment in an adult human).

This study has certain limitations. Translation of rodent data to human clinical situations and applicability is not straightforward. Our data concern mainly segmental motoneuron pools, while in humans, partial loss of motoneuron pools is rather frequent (human injuries rather result in partial losses and less frequently in loss of a single segmental motor pool). Therefore, further clinical research is needed to elucidate the loss of motoneurons in typical injury types and the required number of reinnervating motoneurons after such injuries.

In conclusion, we have here provided evidence that approximately 30% of the original motor pool dedicated to innervate a skeletal muscle is able to maintain the function of a reinnervated recipient muscle at the long term. Further clinical studies are needed to prove the results of this preclinical animal model.

## Data Availability

*No additional data are available.*
